# A fusion approach using GIS, green area detection, weather API and GPT for satellite image based fertile land discovery and crop suitability

**DOI:** 10.1038/s41598-024-67070-1

**Published:** 2024-07-15

**Authors:** Ananthakrishnan Balasundaram, A. B. Abdul Aziz, Aman Gupta, Ayesha Shaik, Muthu Subash Kavitha

**Affiliations:** 1 Centre for Cyber Physical Systems, Vellore Institute of Technology, Chennai, India; 2 School of Computer Science and Engineering, Vellore Institute of Technology, Chennai, India; 3 School of Information and Data Sciences, Nagasaki University, Nagasaki, Japan

**Keywords:** Fertile land discovery, GIS, Crop recommendation, Crop suitability, Computer science, Plant sciences

## Abstract

Proper utilization of agricultural land is a big challenge as they often laid over as waste lands. Farming is a significant occupation in any country and improving it further by promoting more farming opportunities will take the country towards making a huge leap forward. The issue in achieving this would be the lack of knowledge of cultivable land for food crops. The objective of this work is to utilize modern computer vision technology to identify and map cultivable land for agricultural needs. With increasing population and demand for food, improving the farming sector is crucial. However, the challenge lies in the lack of suitable land for food crops cultivation. To tackle this issue, we propose to use sophisticated image processing techniques on satellite images of the land to determine the regions that are capable of growing food crops. The solution architecture includes enhancement of satellite imagery using sophisticated pan sharpening techniques, notably the Brovey transformation, aiming to transform dull satellite images into sharper versions, thereby improving the overall quality and interpretability of the visual data. Making use of the weather data on the location observed and taking into factors like the soil moisture, weather, humidity, wind, sunlight times and so on, this data is fed into a generative pre-trained transformer model which makes use of it and gives a set of crops that are suitable to be grown on this piece of land under the said conditions. The results obtained by the proposed fusion approach is compared with the dataset provided by the government for different states in India and the performance was measured. We achieved an accuracy of 80% considering the crop suggested by our model and the predominant crop of the region. Also, the classification report detailing the performance of the proposed model is presented.

## Introduction

The rapid advancement of technology has opened up new avenues for enhancing agricultural practices^1^. In recent years, the utilisation of satellite imagery has emerged as a valuable tool for analysing and comprehending the Earth's surface. By harnessing the power of computer vision and machine learning techniques, researchers have been able to extract meaningful information from satellite images and apply it to various domains. This paper delves into the employment of satellite imagery and Generative Pre-trained Transformer (GPT) models to identify fertile land and provide precise crop suitability analysis.

Satellite images offer a comprehensive outlook of vast areas, enabling us to observe and analyse the characteristics of different regions. By implementing pre-processing techniques, such as image enhancement and feature extraction, we can extract valuable attributes from these images. For instance, we can detect verdant areas, which often serve as indicators of fertile land suitable for agricultural purposes. By applying a green filter to the satellite images, we can visually highlight the regions adorned with vegetation, thus offering a preliminary understanding of potential cultivable land.

However, prevailing models for crop suggestion and land suitability analysis suffer from certain limitations. Many of these applications rely on antiquated data sources, such as historical climate records and static maps, which may not accurately reflect the current conditions of the land. Furthermore, while GPT models have showcased impressive capabilities in various natural language processing tasks, their application in the realm of crop suitability analysis has remained largely unexplored.

The domain of our research revolves around harnessing the potential of GPT models for meticulous crop suitability analysis. GPT models have demonstrated remarkable performance in generating coherent and contextually relevant text based on input prompts. By extending their application to crop suitability analysis, we strive to provide accurate and up-to-date suggestions based on live data^2^, encompassing factors such as weather conditions, soil moisture, and other pertinent attributes. This approach has the potential to revolutionise the agricultural sector by equipping farmers with valuable insights and recommendations for efficient cultivation practices.

Existing models in this domain often employ convolutional neural networks (CNNs) for image analysis and classification tasks as mentioned in Yang et al.^3^. Also some other works such as Hussain et al.^4^ use Analytical Hierarchy Process (AHP) and Multi-Criteria Decision Analysis (MCDA) techniques to determine land suitability. These models frequently rely on predefined features and patterns, limiting their adaptability to varying conditions and their capacity to generate contextualised recommendations. By incorporating GPT models into the analysis pipeline, we can leverage their ability to comprehend and generate meaningful information from intricate data sources, such as satellite imagery and weather APIs. This integration holds the promise of delivering more accurate and precise crop suitability suggestions.

The motivation behind this work stems from the emerging significance of GPT models and the dearth of research in the realm of efficient farming practices. GPT models have garnered considerable attention in the research community, showcasing their effectiveness across various domains. Nevertheless, their application in agriculture, particularly for crop suitability analysis, remains constrained. By venturing into this uncharted territory, we aim to contribute to the burgeoning body of knowledge in both the fields of generative AI and sustainable farming^5^.

The objective of our work is twofold. Firstly, we strive to develop a robust framework that amalgamates satellite image analysis, weather data, and GPT-based crop suitability analysis to provide accurate and precise crop suggestions. Secondly, we aspire to lay the groundwork for further research in this area, fostering the exploration of advanced techniques and methodologies to enhance agricultural practices.

The significance of our work lies in its potential to revolutionise the farming industry. By furnishing farmers with accurate and data-driven recommendations, we can diminish their reliance solely on intuition and generational knowledge. This paves the way for a future where farmers can make informed decisions based on scientific analysis, ultimately leading to improved crop yields, resource efficiency, and overall sustainability in the agricultural sector.

The following objectives delineate the specific milestones guiding our research endeavour, each tailored to contribute meaningfully to the advancement of knowledge in this domain:

- Formulate a methodological framework aimed at significantly enhancing the quality of satellite imagery from publicly available resources such as Apple Maps or Google Maps.
- Design an algorithm to identify and quantify green areas within the images, calculating the acreage based on the image scale.
- Extract geospatial attributes closely associated with agricultural effects, including soil characteristics, humidity, rainfall, and weather parameters.
- Utilize the artificial generative intelligence model to provide crop suggestions based on the processed data.
- Improve image processing for satellite images through geospatial attributes and convert them into an information extraction worthy image.

## Literature review

Several remarkable research studies have been carried out in the field of GIS-based agriculture, which have greatly influenced the development of this paper. Dornich^6^ presents a comprehensive overview of GIS in agriculture, emphasising its role in spatial analyses and decision-making through visual data representation. Singh et al.^7^ utilise the Analytic Hierarchy Process (AHP) and geospatial techniques to evaluate the suitability of cereal crops in India, with the conclusion that AHP proves effective for such analyses. CIBO Technologies elucidates the importance of remote sensing in agriculture, enabling data-driven interventions to enhance crop yield. Similarly, Moreno-Armendáriz et al.^8^ and Nguyen et al.^9^ employ advanced deep learning algorithms and drone imagery^10,11^ for urban green space analysis, greatly assisting urban planning endeavours. Weatherbit^12^ provides an Agriculture Weather Forecast API, furnishing 8-day weather forecasts specifically tailored for the agriculture industry, thereby supporting informed decision-making by farmers. Rossi et al.^13^ employ a GIS-based geopedological approach to evaluate land suitability for chestnut groves, effectively demonstrating the role of GIS in promoting sustainable rural development.

Siche et al.^14^ explores the multifarious applications of OpenAI and ChatGPT language models in agriculture, whereas Adamchuk et al.^15^ discuss the fundamental principles of agricultural remote sensing and its immense potential for agricultural producers. Mahato et al.^16^ utilise machine learning algorithms to accurately detect and assess green belt areas, while GIS Stack Exchange proposes an efficient method for identifying urban green areas in Google Earth imagery using R, a the popular programming language. Stormglass^17^ provides an Agritech Weather API, delivering highly detailed weather forecasts specifically designed for the agricultural sector. Lastly, Kumar^18^ integrate GIS and machine learning techniques to predict cropland suitability, underscoring the significance of sustainable agriculture. Collectively, these references underscore the immense potential and practical applications of GIS, remote sensing, machine learning, and weather APIs in the exploration and utilisation of fertile land in agriculture. Also, Chang^19^ Real-time weather data, such as cloud computing and a cloud computing building simulation platform Richman et al.^20^, are fundamental components for conducting real-time simulations and making near-future predictions. Du et al.^21^ have devised an approach to forecast the urban heat island effect and indoor overheating in real-time on a city-wide scale. Their work builds on their previous investigations into the dependability of near-future weather data for predicting building performance in the United Kingdom. Accurate weather forecasts play a crucial role in facilitating real-time simulations. In addition to utilising computational fluid dynamics (CFD) models provided by meteorological offices for weather forecasting, satellite technology^22,23^ and camera-based systems^24^ have been employed as weather sensors to predict weather conditions.

With these papers igniting the flame on GIS based research towards agriculture, we are further fuelling the torch with the modern advancements in generative AI that will be quite helpful towards using these existing researches and suggesting the best possible and suitable agricultural produce for the given area of interest.

Climate variability and unpredictable weather patterns further exacerbate the complexity, underscoring the need for adaptive and resilient smart farming practices. In this dynamic landscape, addressing these challenges calls not only for technological advancements but also for a holistic approach that considers the socio-economic realities of farmers^25^, ensuring that the benefits of smart agriculture are realized across diverse agricultural communities. The burgeoning volume of agricultural data presents another challenge, requiring robust analytics and interpretation frameworks to extract meaningful insights. Additionally, the accessibility and affordability of advanced technologies pose barriers for small-scale farmers, necessitating inclusive solutions to ensure equitable benefits^26^.

## Proposed system

**Figure 1:**
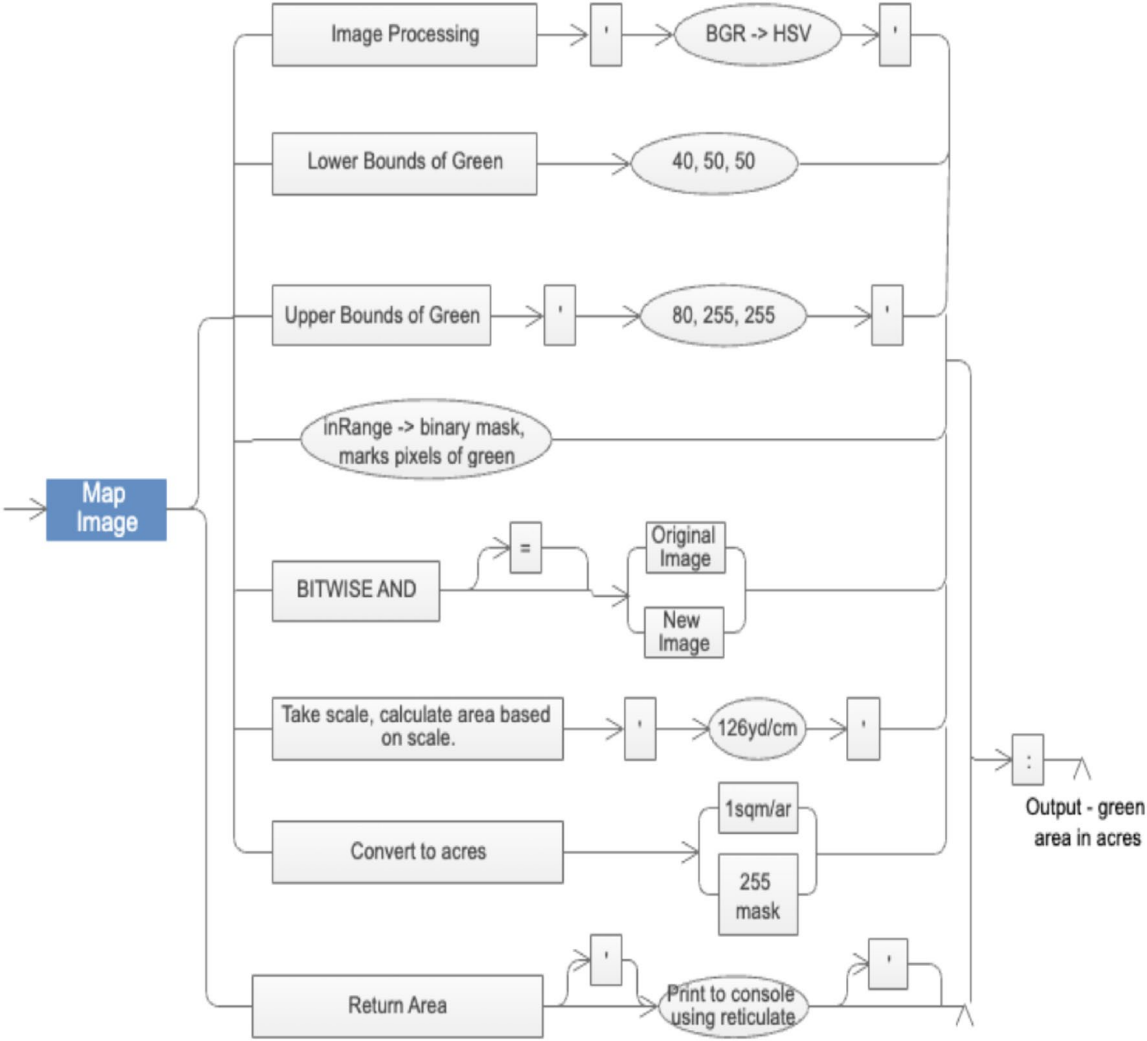
Architecture diagram for proposed satellite image processing.

The structural architecture of the system (Fig. 1) is a unison of different modules working together, it includes from the image procurement to the processing involved in it to enhance it to further make predictions and suitability consulting easier.

### Image collection

To procure the satellite imagery of the region, Apple Maps served as our primary resource. The script employed to compute the area relied extensively on a scaling factor, which was meticulously achieved by zooming into the map until it reached a scale distance of 126 yards/cm. The utilisation of Apple Maps, with its esteemed precision and reliability, proved instrumental in our pursuit of acquiring the satellite imagery necessary for our research, which is then fed into the interface we designed to make the application more user friendly with hopes of launching it as a standalone application as a future work.

### Image processing

The raw image of the area acquired is then passed through an image processing layer that makes it easier to extract the features present in the image, a well known satellite imagery based technique known as the *Panchromatic Sharpening*^27^ is used to sharpen the dull satellite images of various areas, knowing that major satellite view vendors often miss out on providing sharp image details^28^ to the areas of lesser search traffics, it helps in the sub processing layer to make the satellite images more clearer.

**Figure 2:**
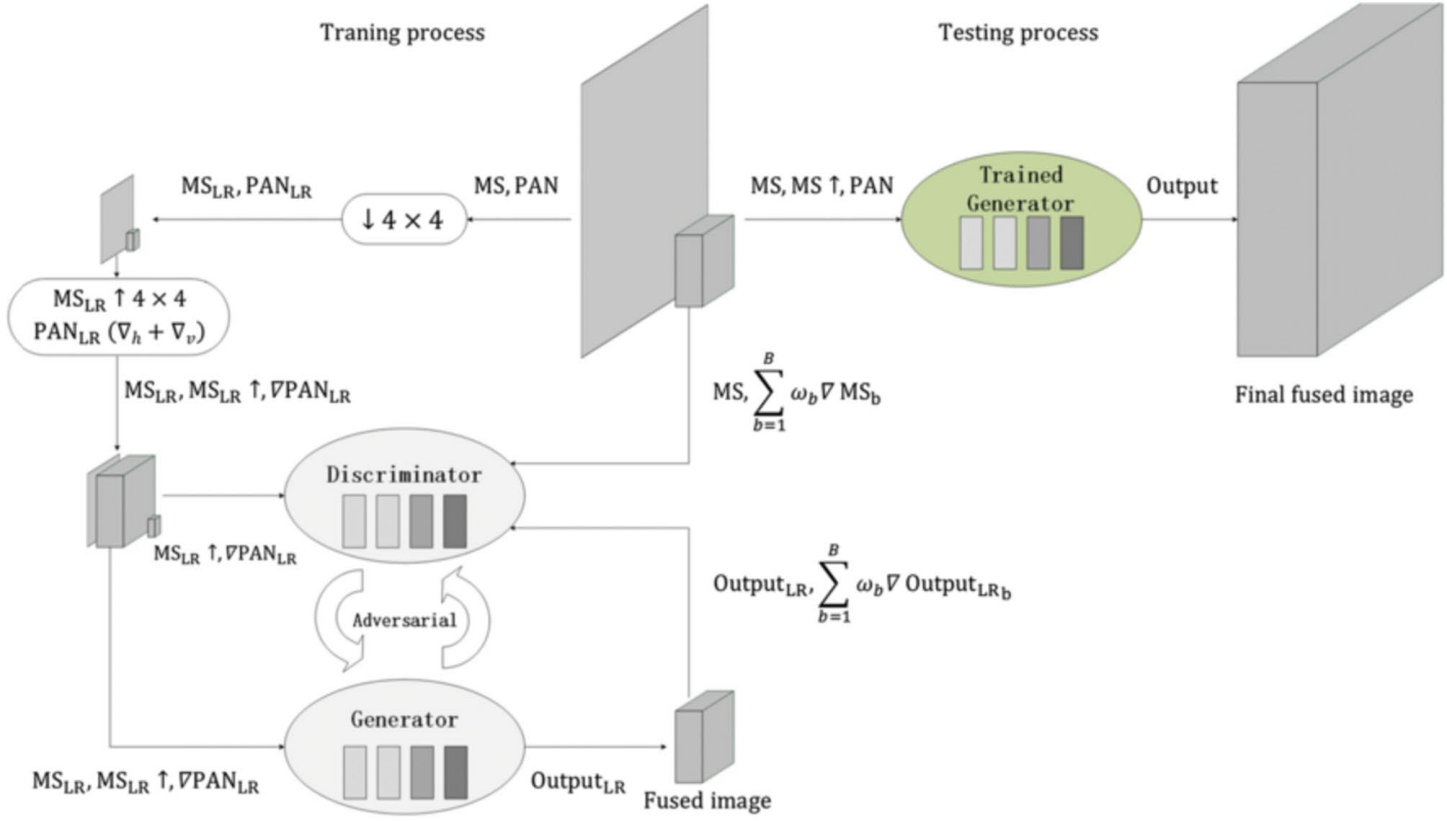
GAN-based panchromatic sharpening.

**Figure 3:**
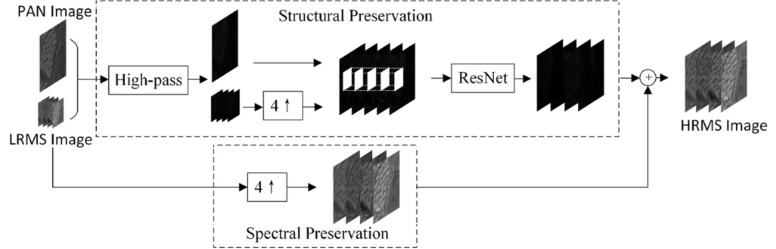
RES-net based panchromatic sharpening.

When it comes to Panchromatic Sharpening^29^ of satellite images, there are usually two methods that are prominent and viable for this application, it includes the generative adversarial network approach (GAN) as depicted in Fig. 2 and a deep network based architecture called the RES-net/PanNet (Fig. 3). Sharpening the images help greatly to make the colours stand out more and have an easier masking procedure to extract the areas out of it.

The boundaries of the buildings are more apparent, which helps to avoid any masking over the buildings and miscalculating the area that is to be found.

#### Conversion of the multispectral image to grayscale

To begin with, the multispectral image is converted to grayscale. This is typically done by taking a weighted average of the red, green, and blue channels, where the weights are determined based on the sensitivity of the human eye to different colours.

#### Resampling the panchromatic image

The panchromatic image, which has a higher spatial resolution, is resampled to match the dimensions of the grayscale multispectral image. This is done to ensure that both images have the same pixel dimensions.

#### High-pass filtering

This involves convolving the image with a high-pass filter kernel, such as a *Laplacian* or a *Difference of Gaussian (DoG)* filter. The high-pass filter enhances the edges and details in the image.

#### Fusion of images

This fusion process is often achieved through a process known as intensity modulation, where the pixel values of the panchromatic image are multiplied with the corresponding pixel values of the grayscale image. We use panchromatic sharpening as its which is quite simple but very effective in our use case as it greatly justifies the quality of the map image. Equation for panchromatic sharpening is given by:

- Let *M* be the grayscale multispectral image,
- *P* be the resampled panchromatic image,
- *F(P)* be the result of applying a high-pass filter to *P*, and
- *λ* be a weighting factor.

1 $$ Sharpened\;image = M + \lambda *F\left( P \right)$$

We are utilising a technique called the Brovey Transformation, which is a fusion-based method commonly employed for combining multiple data sources. It employs a ratio-based algorithm to merge images, assuming that the spectral range of the panchromatic (PAN) image matches that captured by the multispectral (MS) bands. The aim of this transformation is to generate RGB images, merging only three bands at a time. In our approach, we begin by inputting the RGB image, converting it into HSV-based images to obtain a sharper version, and then converting them back using the fusion technique. The Brovey Transformation is mathematically defined by the following equation:

2 $$ {\text{Y}}_{{\text{k}}} \left( {i, j} \right) = X_{{\text{K}}} \left( {i, j} \right)X_{{\text{p}}} \left( {m, n} \right) \div \mathop \sum \limits_{k - 2}^{4} \;X_{{\text{k}}} \left( {i, j} \right) $$

The symbols used in Eq. (2) represent the following:

- X_k_(i,j): Refers to the data of the k_th_ original multispectral (MS) band, with i representing the pixel number and j representing the line number of that specific band.
- Y_k_(i,j): Represents the data of the fused multispectral (MS) band, which is the output of the fusion process. Similar to X_k_(i,j), i and j denote the pixel and line numbers of the fused band.
- X_p_(m,n): Denotes the original panchromatic (PAN) band data, where m represents the pixel number and n represents the line number of the PAN band.

**Figure 4:**
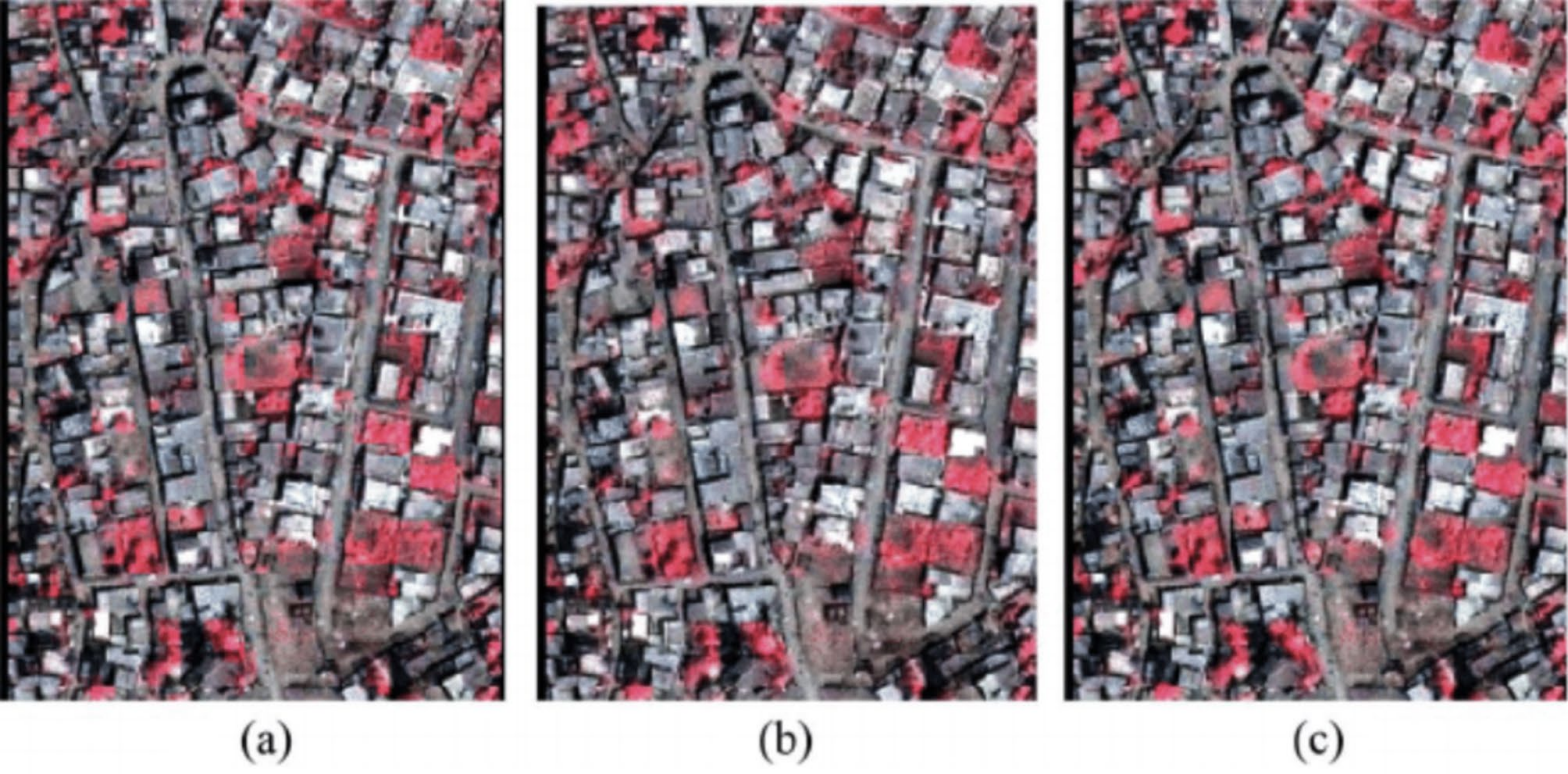
BT image fusion using (**a**) nearest neighbour, (**b**) bilinear interpolation and (**c**) cubic convolution resampling.

The observed outcomes remain visually consistent across all three resampling methods, resulting in a substantial improvement in resolution with smaller pixel values. Notably, there is a noticeable presence of colour distortion, which varies depending on the combinations of bands used as shown in Fig. 4.

From the comparison among other techniques in Table 1, we found out that the Brovey transformation method is quite efficient, although the Principal Component Analysis is almost the same, it is often compute expensive as compared to this with roughly the same results to be noticed in both of them.

**Table 1:** Comparison of other fusion techniques in comparison with the Brovey transformation technique.

Techniques	Building	Trees	Road
Principal component analysis	VG	VG	VG
Multiplicative approach	P	P	P
Brovey transformation	VG	VG	VG
Hyperspherical color space	G	G	G
Modifies IHS	G	P	G
Subtractive	G	G	G
Wavelet transform	P	P	P
HPF	P	P	P
Ehlers	P	G	P

VG, Very Good; G, Good; P, Poor.

### Colour masking, segmentation and area calculation

To extract the green area from a map image, a thresholding technique can be applied after converting the image from the RGB colour space to the HSV colour space. This approach allows for capturing varying intensities of green more effectively compared to using a filter in an RGB setup. The HSV colour space separates the colour information into three components: Hue, Saturation, and Value. In this case, we are particularly interested in the Hue component, as it represents the dominant colour information. The steps involved in this process are as follows:

1. Convert the image from RGB to HSV colour space, this conversion is performed to represent the image in terms of Hue, Saturation, and Value. Each pixel's RGB values are transformed into corresponding HSV values using Eq. (3).
2. Thresholding based on Hue values, define a lower threshold and an upper threshold to identify the desired green spectrum. These thresholds should encompass the range of green hues you want to extract. For example, a lower threshold could be set to a lighter green, while the upper threshold could be set to a darker green. These thresholds depend on the specific shades of green you are interested in.
3. Apply the thresholding operation, compare the Hue values of each pixel in the HSV image with the defined lower and upper thresholds. If the Hue value falls within this range, set the pixel value to white (indicating the green area); otherwise, set it to black (indicating non-green areas).
4. Obtain the green area, the resulting thresholded image will have white regions where the green areas are detected and black regions for non-green areas. The white regions represent the extracted green area from the map image.

RGB to HSV Conversion equations are given as follows:

- Color cone

$$ \begin{aligned} & H = hue{/}color\;in\;degrees \in \;\left[ {0,360} \right] \\ & S = saturation\; \in \;\left[ {0,1} \right] \\ & V = value\; \in \;\left[ {0,1} \right] \\ \end{aligned} $$

- Conversion RGB → HSV

3 $$\begin{aligned} & V,\;max = max\left( {R,G,B} \right),\;min = min\left( {R,G,B} \right) \\ &S = \left( {max - min} \right)/max\; \left( {or \, S = 0,\;if\;V = 0} \right) \\ &H = 60*\left( {0 + \left( {G - B} \right)/\left( {max - min} \right)} \right),\;\;if \, max = R \\ & \quad = 60*\left( { \, 2 + \left( {B - R} \right)/\left( {max - min} \right)} \right),\;if\;max = G \\ & \quad = 60*\left( { \, 4 + \left( {R - G} \right)/\left( {max - min} \right)} \right),\;if\;max = B \\ &H = H + 360,\;if\;H < 0 \\ \end{aligned} $$

The HSV image is then extracted and the area is calculated based on the number of acres per centimetre of the scaled image which is fixed to a scale of 126 yards/cm.

### Getting geospatial characteristics

In order to gather geospatial attributes about a particular location, we employ the use of X and Y APIs. These APIs provide us with valuable data pertaining to botanical growth factors such as soil moisture, temperature, weather attributes, humidity, and soil characteristics, among others. By utilising this information, we establish a foundation for our GPT (Generative Pre-trained Transformer) model to acquire knowledge regarding the area's weather and soil conditions. These geospatial attributes are collected and inputted into the GPT model. The user simply uses the interface and types the name of the location, on submitting, the API calls are made and the data is procured.

### Feeding the geospatial characteristics into the generative pre-trained transformer

The data collected by the API pipeline is then passed through the transformer model, specifically the *davincii-002* variant, which enables us to predict potential crop outcomes feasible for cultivation in the given location under the specified conditions. To ensure accuracy, we cross-reference our findings with the Crop Weather Calendar^30^, a comprehensive dataset compiled by the Indian Council of Agricultural Research, encompassing information about various crops and their respective growth conditions. Table 2 provides information of corp wise CWC from All India Coordinated Research Project on Agrometeorology (AICRPAM) centres.

**Table 2:** Information of Crop wise CWC from AICRPAM centres.

Name of the districts (centres)	Crops
Bangalore, Karnataka	Groundnut
Hisar, Haryana	Mustard
Bhubaneswar, Orissa	Rice
Kanpur, UP	Rice
Jabalpur, MP	Soybean
Raipur, Chattisgarh	Wheat
Udaipur, Rajasthan	Wheat
Parbhani, Maharashtra	Cotton

Notably, the crops identified within the research area as generated by the GPT model align with the ones documented in the Crop Weather Calendar. Moreover, the model also suggests additional crop varieties that have not been explored in the research but are deemed viable based on the provided conditions. These novel crops represent possibilities for cultivation in the area and hold potential without encountering significant obstacles.

The primary objective of this research is to identify suitable crops that can be grown on a specific piece of land, considering the prevailing conditions. Furthermore, we aim to discover new crop varieties that have not been previously experimented with. Instead of relying on traditional trial and error methods, we leverage the advancements of artificial intelligence to predict outcomes based on learned patterns and outcomes.

**Figure 5:**
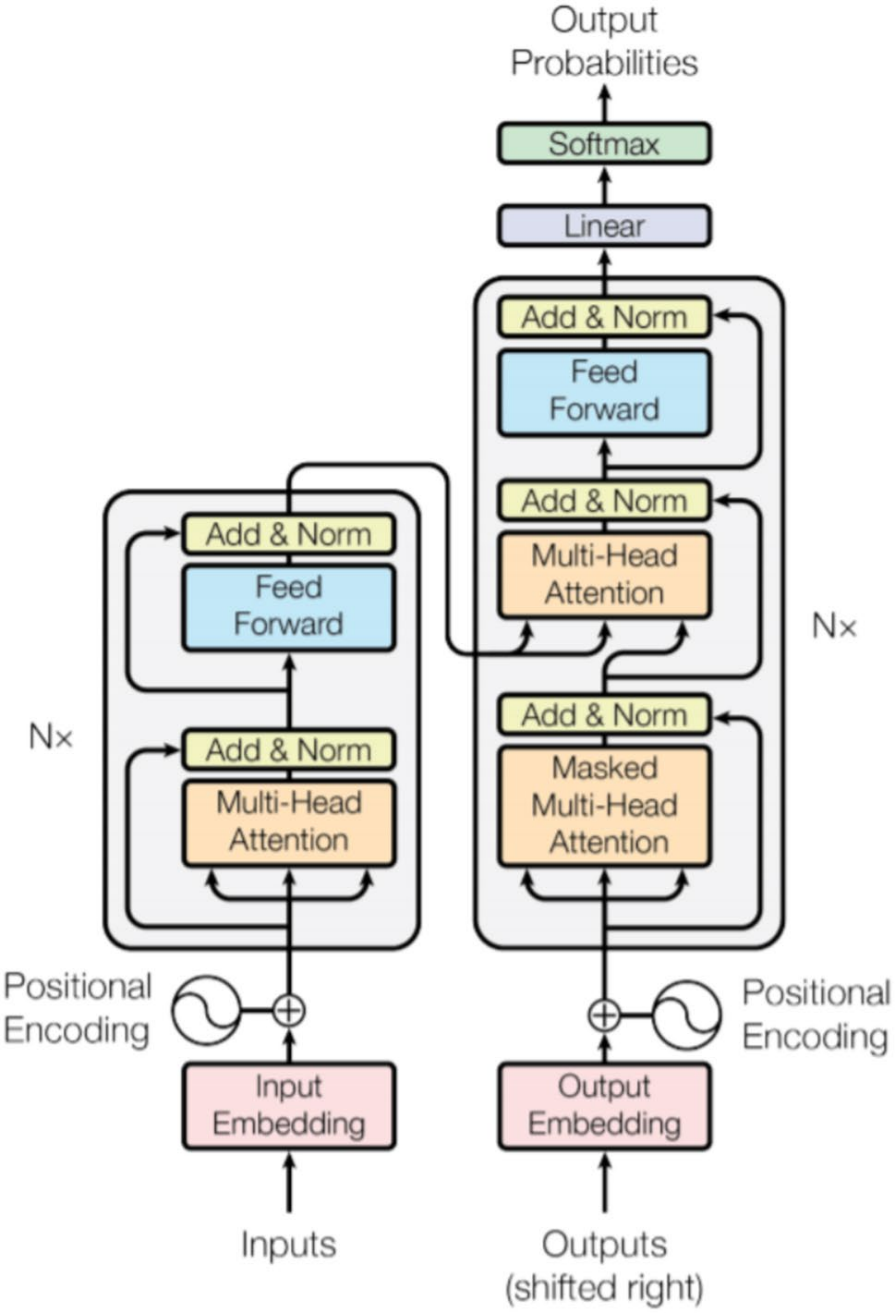
A basic transformer model architecture.

The transformer architecture (Fig. 5) comprises an encoder and a decoder. It excels in modelling long-range dependencies and parallel computations. With self-attention as its crown jewel, transformers have become the cornerstone of language models like GPT and BERT, enabling enhanced language understanding and generation capabilities. Residual connections and layer normalisation fortify training, propelling transformers to surpass recurrent neural networks. In essence, transformers usher in a new era of linguistic finesse and innovation.

The generative capabilities of these models can be used to reason out what crops to grow at a given place using the attributes fed into it, thanks to the countless amounts of data and hours spent training this model with tons of information.

There are various models that exist within, the one used mainly in this research is the GPT-4 model, which is the cutting edge model as of this writing. The workflow of the model consists of classification, entailments, similarity and decision of multiple choices.

**Figure 6:**
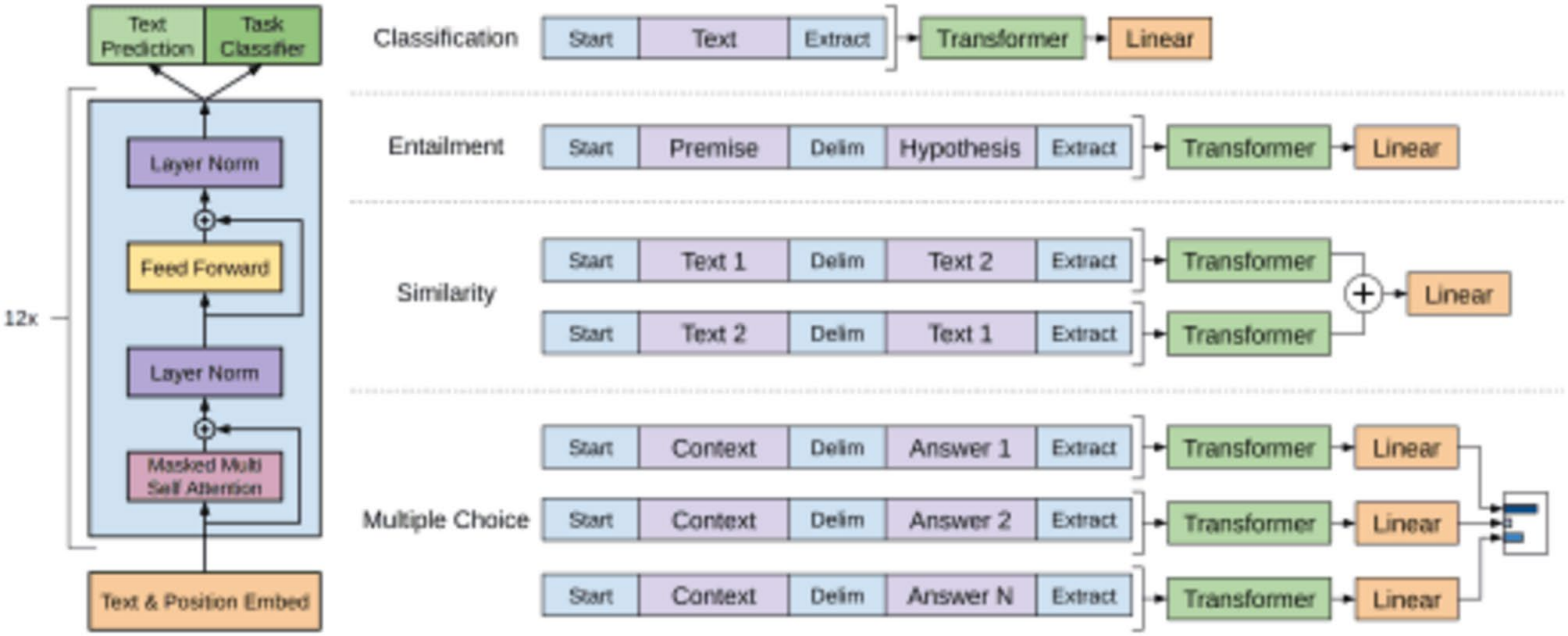
Decision process of the transformer model.

Once the encoding process is over, the task-specific layers are employed based on following objectives as depicted in Fig. 6:

1. Classification: For classification tasks, such as crop analysis or crop categorization, a classification layer is added on top of the encoder. This layer maps the contextualised representations of the input sequence to the appropriate class labels.
2. Entailment: The goal here is to determine the logical relation between the two data points from the API, whether one attribute entails or contradicts the other, is neutral with respect to another etc.
3. Similarity: It involves measuring semantics between the data points, how they affect each other, scoring mechanisms are used to find the similarities.
4. Multiple-Choice Decision: This involves scenarios where multiple crops can be grown, each choice is processed separately through an encoder and task-specific layers, the representation is then compared or scored against each other to make the final outcome.

**Figure 7:**
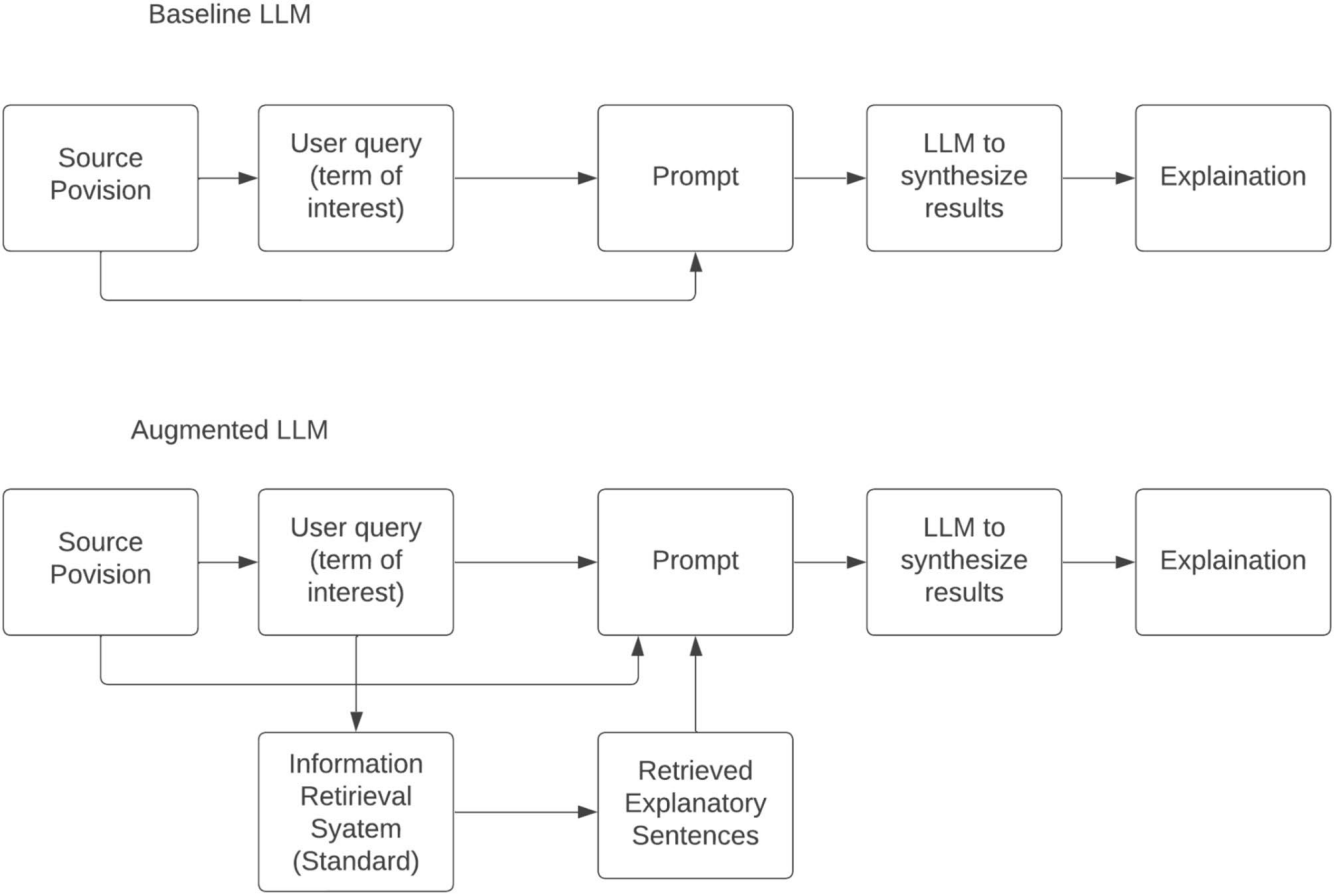
Workflow of the transformer model.

A baseline Large Language Model (LLM) assumes the role of the foundational iteration, devoid of alterations. It dutifully acquaints itself with language patterns derived from copious data sources. Conversely, an augmented LLM transcends the ordinary. Bolstered by grander dimensions, domain-specific training, and supplementary data, it adroitly captures the subtleties of language as depicted in Fig. 7. Purposeful modifications, tailored to specific tasks, refine its performance, thus endowing augmented models with unparalleled proficiency in comprehending and crafting linguistic expressions.

### Decision making by GPT-4 model by considering the input attributes

**Figure 8:**
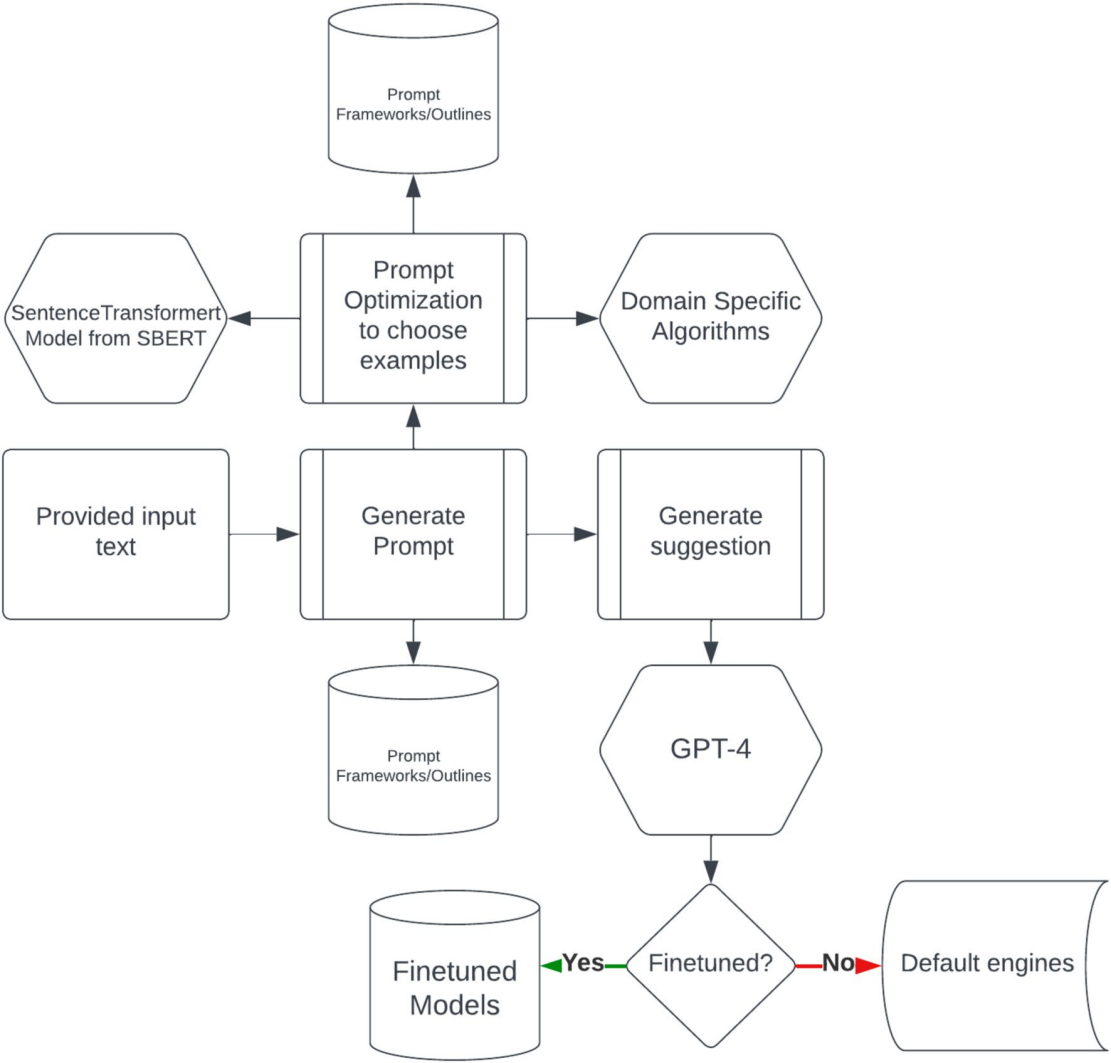
Overview on the reasoning of crop suitability analysis.

The decision process of GPT-4 (Fig. 8) while deciding which crops to suggest is done by analysing the weather conditions, starting with the temperature. If the temperature exceeds 30 °C, the model takes into account the humidity level. When the humidity is low, it suggests crops like Groundnut, Sunflower, Millets, and Cotton. On the other hand, if the humidity is high, it recommends crops such as Rice and Sugarcane. In the case of moderate temperatures (20–30 °C), the model considers the humidity level once again. It suggests crops like Rice, Ragi (Finger Millet), Maize, Pulses such as pigeon pea (Toor Dal) and chickpea, vegetables like tomato, brinjal, and okra, and fruits like mango, banana, and guava. It also suggests considering Coffee and Tea in appropriate hilly regions.

Afterward, it takes into account the wind speed. If the wind speed is high, it proposes selecting crops with strong root systems and wind-resistant characteristics. Conversely, if the wind speed is low, a broader range of crops can be considered. It then evaluates the precipitation levels. When there is high precipitation, it recommends crops like Rice and Sugarcane that require more water. However, when precipitation is low, it suggests considering drought-resistant crops such as Millets, Groundnut, and Sunflower.

Additionally, it considers the UV index. In the event of a high UV index, it suggests opting for crops that can withstand high levels of UV radiation. On the contrary, when the UV index is low, a wider variety of crops can be considered. Once the weather conditions have been assessed, the model moves on to examine the soil conditions, specifically the soil moisture. If the soil moisture is high, it suggests crops that require more water, including Rice, Sugarcane, and fruits like mango, guava, and banana. Conversely, if the soil moisture is low, it recommends considering drought-resistant crops such as Millets, Groundnut, and Sunflower.

### Obtaining the crops and comparing against the crop weather calendar

The obtained results are then compared with the CWC (Crop Weather Calendar) to get accuracy metrics of existing crops that are grown in certain regions and also, the new crops that can be grown with these series of crops that are already widely grown. Furthermore, this analysis unveils novel prospects for cultivating complementary crops, thus broadening the repertoire of agricultural endeavours in the hallowed fields of innovation and sustainability.

Comparing the results with the government dataset made it possible to deduce some conclusions from the accuracy of GPT-4 in predicting the crop suitability of the particular area. This is often a greater step towards improving agricultural conscience in making sure more suitable crops are grown in areas where it previously wasn’t grown.

## Experimental results

### Experimental setup

The research was conducted using a hardware setup consisting of an 8-core Apple M1 processor and 16 GB RAM. To facilitate the execution of GPT instances, Microsoft Azure Cloud Computing was employed. The development environment involved the utilisation of software tools such as Visual Studio Code and RStudio. The Azure AI Platform served as a pivotal component in the research workflow. Programming languages including Python, R, and associated libraries such as OpenCV, RShiny, and huggingface transformers were employed for image processing, data analysis, and model implementation. WeatherAPI, Agro Monitoring, Stormglass, and the dataset provided by the Indian Council of Agricultural Research Crop Weather Calendars were integral sources of information for the research.

### Experimental results

**Figure 9:**
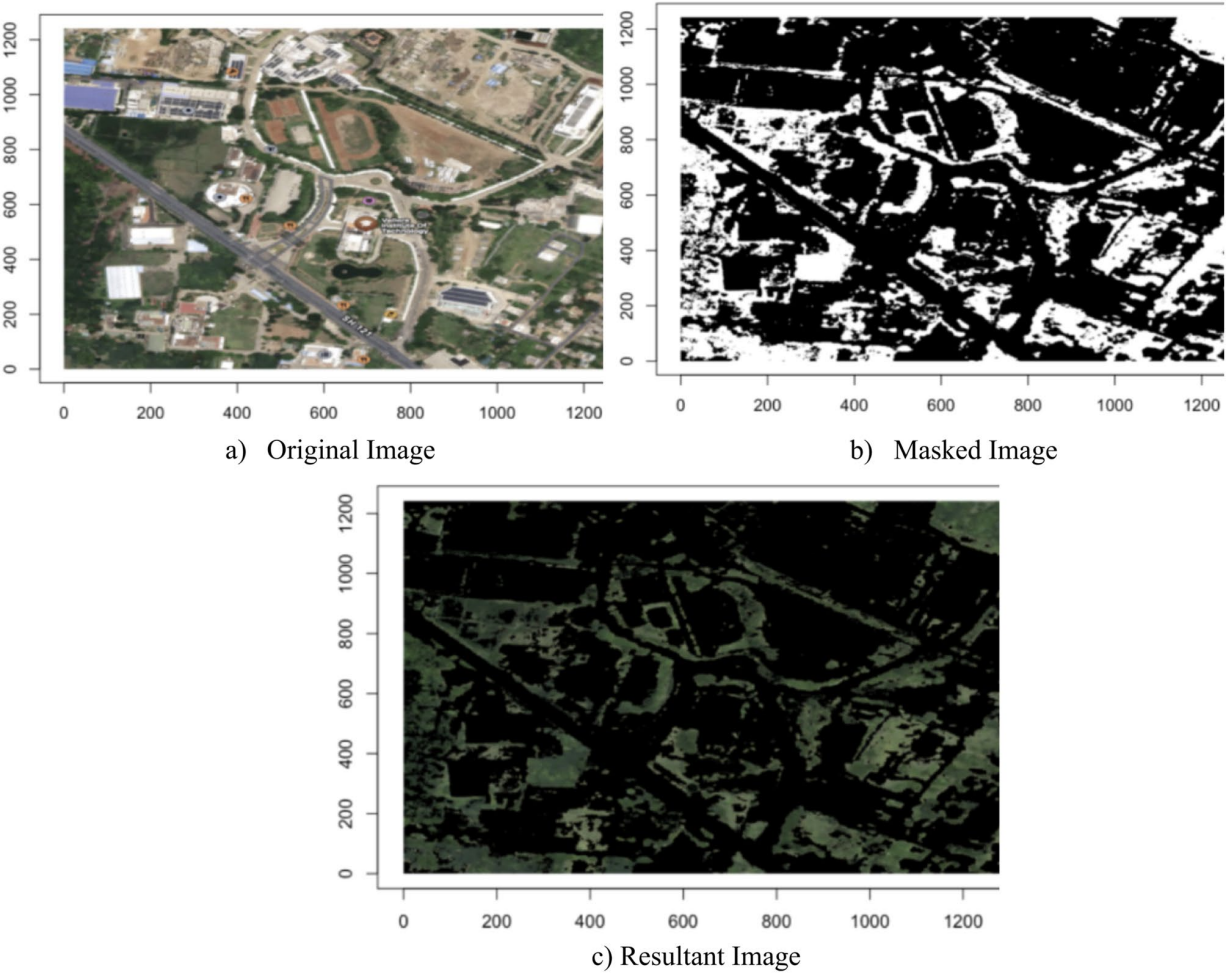
Image acquisition and feeding.

Figure 9 illustrates the results of the image mapping process, with the primary objective of removing extraneous elements like buildings and roads from the scene, thus honing the focus exclusively on the green areas of interest. This feat is accomplished through the adept application of a negation mask, which skillfully extracts the upper and lower bounds of the green hue, subsequently permitting the subtraction of these precise regions from the original captured image. Consequently, this method deftly isolates and accentuates the green regions within the image, elegantly aligning with the intended focus of concern.

**Figure 10:**
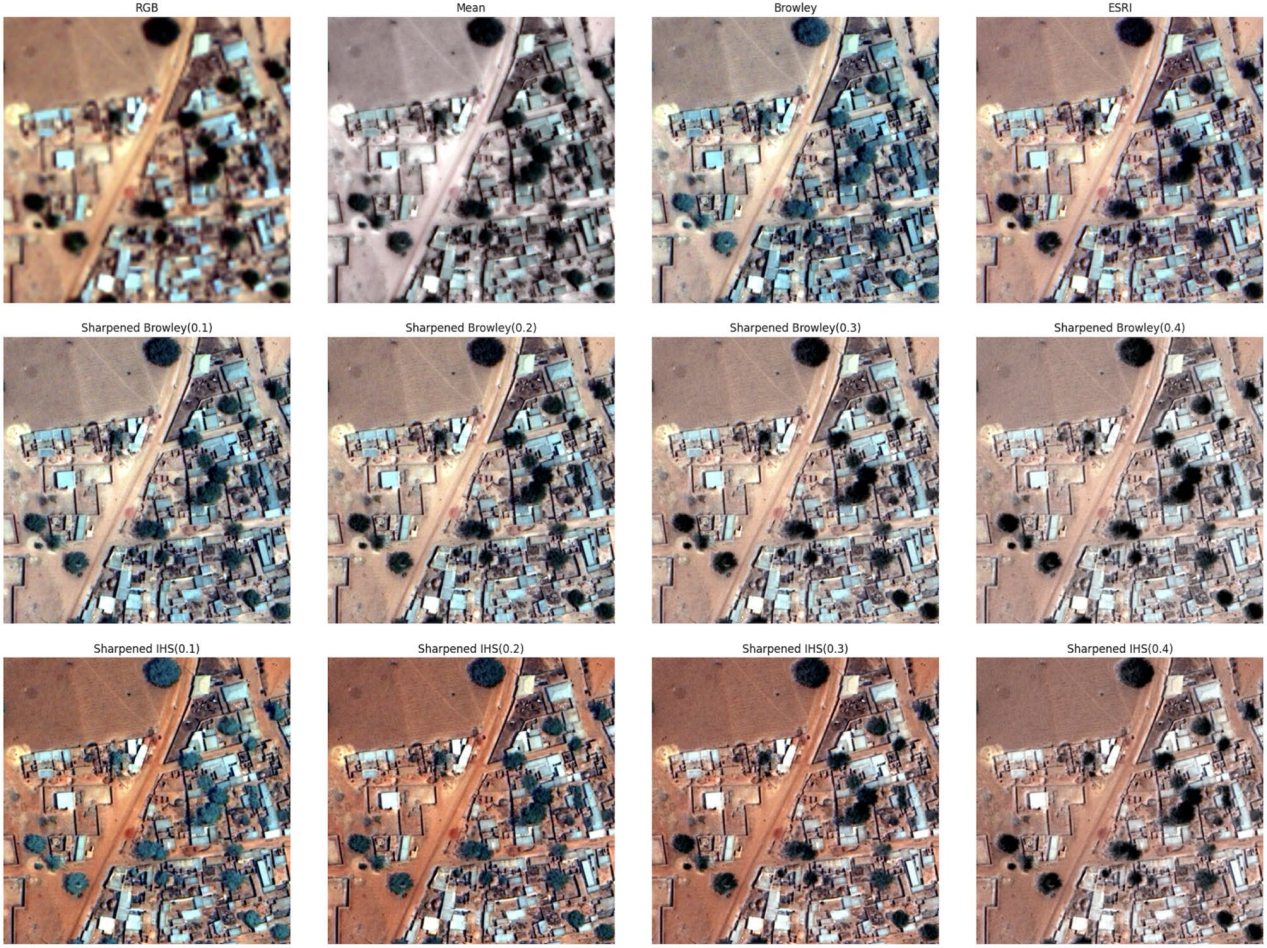
Panchromatic sharpening.

The filtered panchromatic image is then combined with the grayscale multispectral image to create a sharpened image. The resampled panchromatic image is then subjected to a high-pass filtering operation. This significantly sharpens the dull satellite images that are available to use and makes edge detection for buildings and roads more prominent, thereby, making us achieve maximum accuracy in the image processing section to calculate the acres of land available as green areas to be cultivated (Fig. 10).

**Figure 11:**
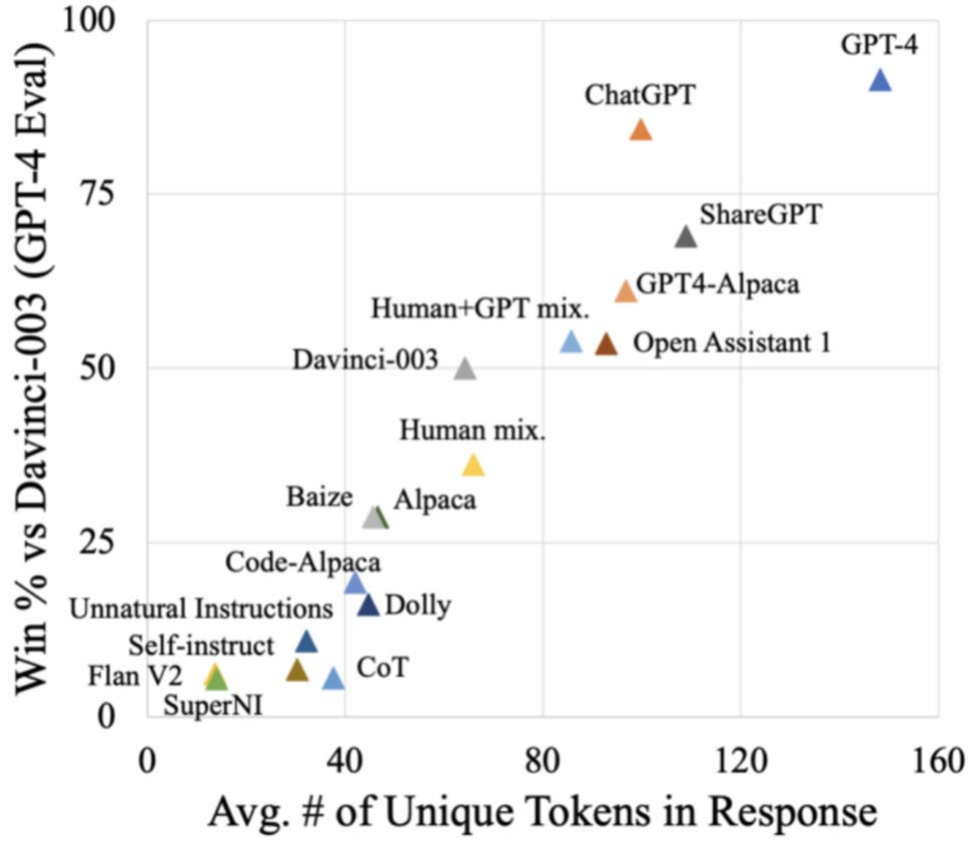
GPT-4 compared against other equivalent models.

Figure 11 portrays the win rate scores of 13b models given by GPT-4, correlates with the average number of unique tokens in the responses, the pearson constant, r is 0.96.

**Figure 12:**
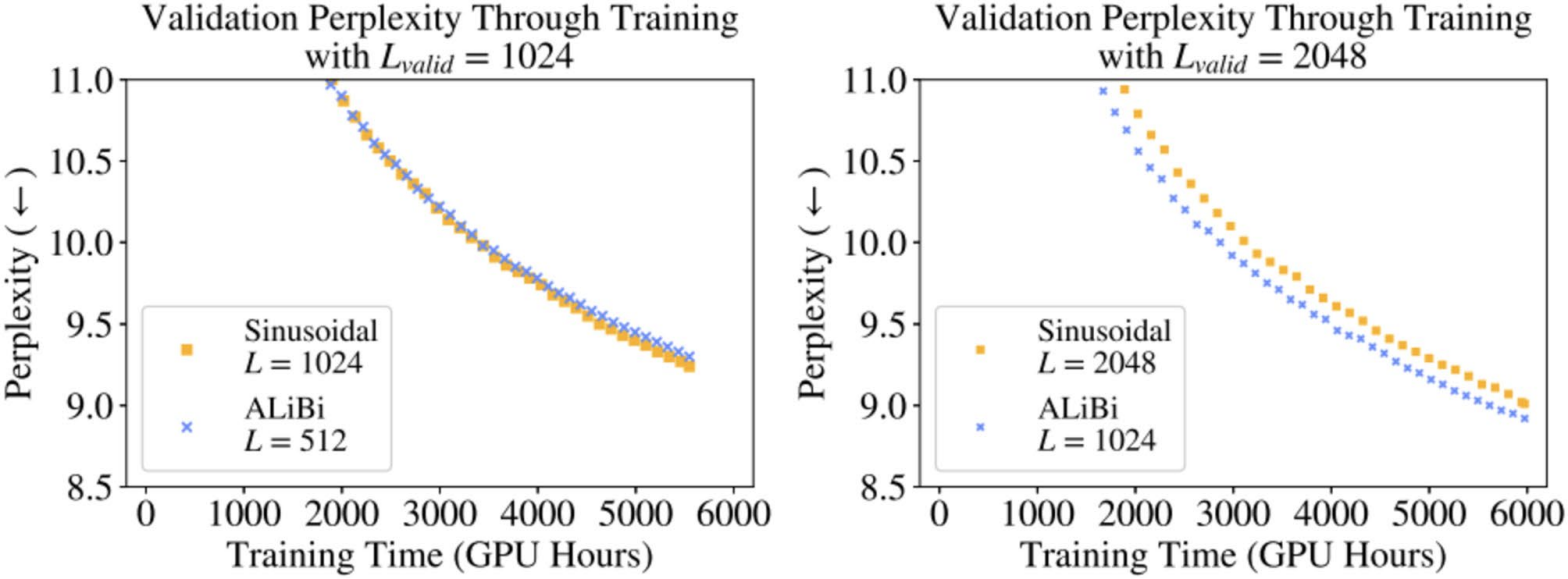
Sinusoidal versus ALiBi models with varying perplexity and training times.

The training time of these models is a factor that affects the responses greatly, however, the perplexity, although doubled, won’t really affect the models despite having half the training size (Fig. 12).

To validate the test cases, we applied our model into multiple districts across India and checked the results with the Crop Weather Calendar. In pretty much all the cases, our model successfully predicted the predominant crop for the respective districts and proved what it's capable of. Although this is ideal, sometimes it was found that the predominant crop was not ranked at the top, rather, at the second or third position; despite this being a minor variation to look for as ultimately the crops shown are the ones that can be grown, the reason for this is attributed to various factors such as local variations in the state region as the dataset is focused entirely on a certain part of the state; another factor could be the limitation of the model’s training but it is a very slim margin on that. Regardless of what it may be, the model consistently provided accurate predictions for the predominant crop, aligning with the guidelines set by the CWC.

**Figure 13:**
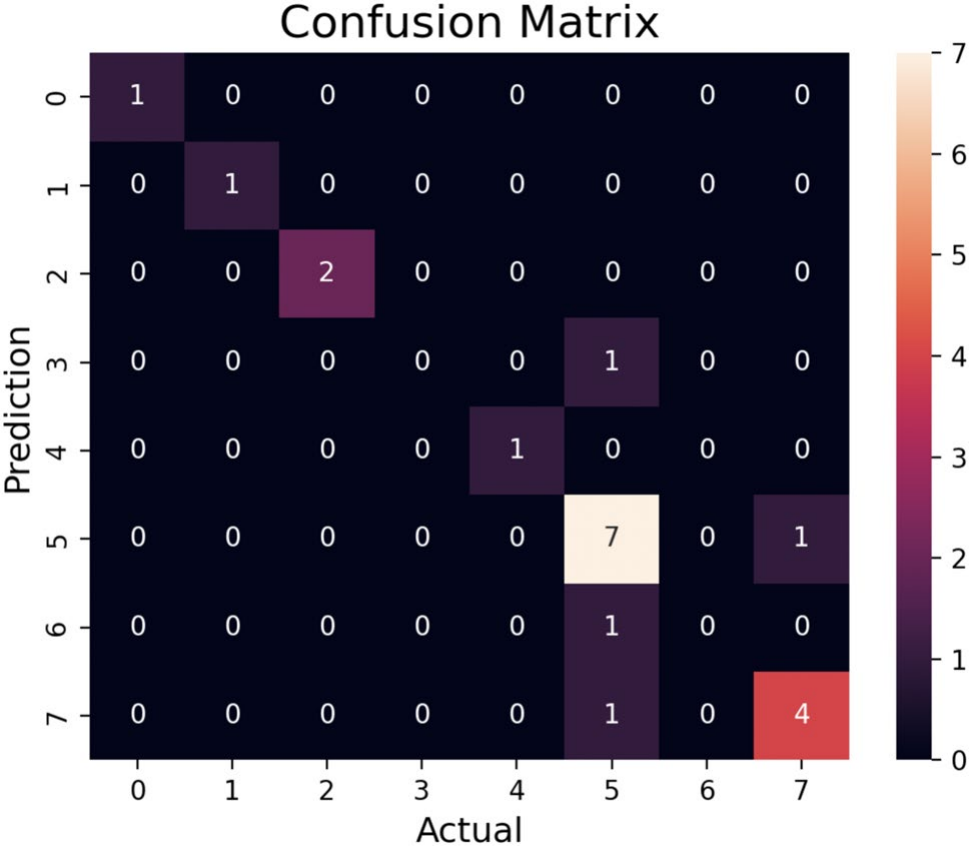
Confusion matrix of crop suitability analysis.

In the confusion matrix (Fig. 13), each cell represents the count of predictions for a specific combination of true label and predicted label. For example, the value 7 in the cell corresponding to “Rice” as the true label and “Rice” as the predicted label indicates that there were 7 instances where the model correctly predicted rice as the predominant crop. Results of the evaluation metrics are illustrated in Table 3. Equations for the Evaluation Metrics used are follows:

- TP = True Positive
- FP = False Positive
- TN = True Negative
- FN = False Negative

4 $$ \begin{aligned}  Precision &= TP/\left( {TP + FP} \right) \\  Recall &= TP/\left( {TP + FN} \right) \\  F1\;score &= \left( {Precision*Recall} \right)/\left( {Precision + Recall} \right) \\ \end{aligned} $$

**Table 3:** Classification report.

Crops	Precision	Recall	F1-Score	# of Instances
Chickpea	1.00	1.00	1.00	1
Cotton	1.00	1.00	1.00	1
Groundnut	1.00	1.00	1.00	2
Maize	0.00	0.00	0.00	1
Mustard	1.00	1.00	1.00	1
Rice	0.70	0.88	0.78	8
Soybean	0.00	0.00	0.00	1
Wheat	0.80	0.80	0.80	5

Table 4 represents the comparison of the results predicted by proposed system with the dominant crop provided in the dataset. The 0’s represent the instances where the dominant crop isn’t in the first position among the predicted crops; and the 1’s represent the converse.

**Table 4:** Predicted versus dominant crop (provided dataset).

Districts	Dominant crops (provided dataset)	Predicted crops	Result
Udaipur, Rajasthan	Wheat	Wheat	1
Raipur, Chattisgarh	Wheat	Rice	0
Palampur, HP	Wheat	Wheat	1
Anand, Gujarat	Wheat	Wheat	1
Jabalpur, MP	Soybean	Rice	0
Faizabad, UP	Chickpea	Chickpea	1
Parbhani, Maharashtra	Cotton	Cotton	1
Anantapur, AP	Groundnut	Groundnut	1
Bangalore, Karnataka	Groundnut	Groundnut	1
Jammu, J&K	Maize	Rice	0
Hisar, Haryana	Mustard	Mustard	1
Ranchi, Jharkhand	Rice	Rice	1
Ludhiana, Punjab	Wheat	Wheat	1
Thrissur, Kerala	Rice	Rice	1
Jorhat, Assam	Rice	Rice	1
Kanpur, UP	Rice	Wheat	0
Dapoli, Maharashtra	Rice	Rice	1
Bhubaneswar, Orissa	Rice	Rice	1
Mohanpur, UP	Rice	Wheat	0
Kovilpatti, TN	Rice	Rice	1

For example, in District A, rice was identified as the predominant crop by our model, which coincided with the recommendations provided by the CWC. The employed satellite image processing techniques involved extracting vegetation indices, analysing soil moisture levels, temperature patterns, wind speed, and humidity. By combining these attributes, our model effectively assessed the suitability of rice cultivation in this district.

Likewise, in District B, our model accurately recognized wheat as the predominant crop. The image processing techniques utilised encompassed the analysis of various spectral bands, calculation of vegetation indices, and evaluation of soil moisture content. The model's output was in alignment with the CWC, thus empowering farmers with precise information to make informed decisions.

One significant aspect of our model is its ability to propose complementary crops alongside the predominant crop for a given region. We achieved an accuracy of 80% considering the crop suggested by our model and the predominant crop of the region. This feature grants farmers a broader spectrum of choices, empowering them to exercise greater control over their crop selection.

## Discussion

The crux of this research lies in the convergence of Geographical Information Systems (GIS), satellite imagery, weather APIs, and Artificial Generative Intelligence (AGI) models to revolutionize crop suitability analysis in agriculture. By considering relevant attributes and environmental factors, our model suggests crops that have the potential to thrive in the region, surpassing the confines of traditional crop options. This aspect enables farmers to diversify their crop portfolio and make informed decisions regarding the suitability of crops specific to their region. The limitations of prevailing models become evident when considering factors such as reliance on antiquated data sources like historical climate records and static maps. AGI models, known for their prowess in natural language processing, remain relatively unexplored in the realm of crop suitability analysis. This research seeks to bridge this gap by harnessing the potential of AGI/GPT models for meticulous crop suitability analysis.

Moreover, our model extends beyond the identification of predominant crops and extends its scope to propose supplementary crop options. By considering relevant attributes and environmental factors, our model offers recommendations for additional crops that show potential for thriving in the given region. This valuable information empowers farmers to exercise greater control over their crop selection, facilitating the diversification of their crop portfolio and enabling them to make well-informed decisions regarding suitable crops specific to their region. This aspect grants farmers the opportunity to explore novel crop alternatives, adapt to changing market demands, and potentially enhance their profitability. It can be observed from Table 3 that the classification report gave good precision, recall and F1-score for most of the crops. Also, Table 4 asserts that out of 20 crops considered under study, the proposed model prediction matched the dominant crop as provided in the dataset 15 times underlining the consistency is predicting the right crop for the region.

Through the integration of sophisticated image processing techniques and leveraging the capabilities of our GPT-based model, we provide farmers with a comprehensive analysis of their agricultural lands. The supplementary crop suggestions furnished by our model present farmers with a wider array of choices, augmenting their decision-making process. This not only fosters a greater sense of autonomy for farmers in selecting the crops they wish to cultivate but also assists them in making informed choices regarding crops that are best suited for their particular region. Consequently, this approach promotes sustainable farming practices and cultivates a culture of agricultural innovation.

## Conclusion and future work

In this research, we have proposed a GIS-based system that leverages satellite images, green area detection, weather APIs, and GPT crop suitability analysis to facilitate the discovery of fertile land and provide accurate crop recommendations. Our unique approach combines location-based analysis with the integration of soil and climate attributes, enabling us to offer tailored suggestions for crop cultivation. Additionally, our system possesses the capability to utilise predicted climate data, allowing us to provide recommendations for future dates. One of the key differentiating factors of our system is the utilisation of location information. By incorporating location data, we can pinpoint specific regions and analyse the soil and climate attributes of those areas. Satellite imagery, a cornerstone of this research, provides a panoramic view of extensive areas, enabling comprehensive analysis of different regions. Leveraging pre-processing techniques, such as image enhancement and feature extraction, allows for the extraction of valuable attributes from satellite images. The proposed model reported a good precision, recall and F1-score for most of the crops. Also, the prediction of dominant crop by the model matched with the dominant crop provided in the dataset in 15 out of 20 instances. By empowering farmers with data-driven insights, the proposed system aims to reduce reliance on intuition and generational knowledge, paving the way for informed decisions, improved crop yields, resource efficiency, and overall sustainability in agriculture. The research signifies a bold step towards the future of agriculture, where cutting-edge technology plays a pivotal role in shaping efficient and sustainable farming practices.

Future work will be towards enhancing the accuracy of identifying cultivable land. Currently, we utilise a basic green area detection mechanism, which may include non-agricultural spaces such as buildings, small parks, or backyards. To address this, we can explore machine learning algorithms to differentiate between suitable agricultural land and non-cultivable areas. By training the model on a diverse range of agricultural data and incorporating domain-specific knowledge, we can enhance its ability to provide more accurate and contextually relevant recommendations. By expanding the system to include additional factors such as market demand, economic viability, and crop rotation can provide farmers with a more comprehensive and holistic decision-making tool.

## Data Availability

The dataset with which the predicted results have been compared in Table 4 is available in http://www.cropweatheroutlook.in/crida/amis/AICRPAM%20Bulletin%20(District%20Level%20Wthr%20Calendars).pdf^30^.
